# Designing for Confidence: The Impact of Visualizing Artificial Intelligence Decisions

**DOI:** 10.3389/fnins.2022.883385

**Published:** 2022-06-24

**Authors:** Alexander John Karran, Théophile Demazure, Antoine Hudon, Sylvain Senecal, Pierre-Majorique Léger

**Affiliations:** HEC Montréal, Université de Montréal, Montreal, QC, Canada

**Keywords:** explainability, confidence, trust, HCAI, cognitive fit, decision support

## Abstract

Explainable artificial intelligence aims to bring transparency to artificial intelligence (AI) systems by translating, simplifying, and visualizing its decisions. While society remains skeptical about AI systems, studies show that transparent and explainable AI systems can help improve the Human-AI trust relationship. This manuscript presents two studies that assess three AI decision visualization attribution models that manipulate morphological clarity (MC) and two information presentation-order methods to determine each visualization’s impact on the Human-AI trust relationship through increased confidence and cognitive fit (CF). The first study, *N* = 206 (Avg. age = 37.87 ± 10.51, Male = 123), utilized information presentation methods and visualizations delivered through an online experiment to explore trust in AI by asking participants to complete a visual decision-making task. The second study, *N* = 19 (24.9 ± 8.3 years old, Male = 10), utilized eye-tracking technology and the same stimuli presentation methods to investigate if cognitive load, inferred through pupillometry measures, mediated the confidence-trust relationship. The results indicate that low MC positively impacts Human-AI trust and that the presentation order of information within an interface in terms of adjacency further influences user trust in AI. We conclude that while adjacency and MC significantly affect cognitive load, cognitive load alone does not mediate the confidence-trust relationship. Our findings interpreted through a combination of CF, situation awareness, and ecological interface design have implications for the design of future AI systems, which may facilitate better collaboration between humans and AI-based decision agents.

## Introduction

Artificial intelligence (AI) and machine learning algorithms are increasing in sophistication and accuracy to automate an ever-increasing array of tasks. However, these algorithms’ rapid growth in complexity and performance makes them more opaque and less interpretable. Thus, the decisions taken and processes used by these AI systems to make said decisions are more challenging to understand and explain for its end users (Rudin, 2019).

Within the field of AI, interpretability can be defined as “the ability to explain or to present in understandable terms to a human” (Doshi-Velez and Kim, 2017). It represents the degree to which a human can understand the basis of a decision or the extent to which a human can predict a machine learning model’s result with a high degree of consistency. The more interpretable the model, the easier it is to understand why certain decisions or predictions have been made. This relationship also engenders confidence between the user of a decision support engine and the machine learning algorithm that forms and then provides those decisions.

In this work, we report on a study that aims to assess the effect of interpretability techniques and visualizations on user confidence in the AI system providing decisions. Current research in the field focuses more on creating mathematically interpretable models, neglecting the human who uses these explanations (Abdul et al., 2018). Taking a user-centric approach to interpretability and designing to foster confidence may better facilitate collaboration between humans and AI decision systems and assuage societal concerns around the use of AI (Bryson and Theodorou, 2019). This study attempts to bridge this gap in interpretability by providing human-centered explanations using various techniques without compromising the faithfulness of the AI visualization.

Research in explainable AI (XAI) seeks to create interpretability models and methods to address the problems associated with a lack of transparency in AI systems, therefore making them more explainable and fairer (Barredo Arrieta et al., 2020). Some methods developed for image recognition systems attempt to highlight which components of the AI model or system are perturbed to create decisions. Visualizations or descriptions of discriminative mechanisms are then used to represent perturbed components of the system for end-users. These methods are essential to identify sources of potential bias in the training data and ensure that algorithms perform as expected (Gilpin et al., 2018). Providing explanations of system behaviors as a form of transparency has been shown to have a considerable positive impact on developing confidence in new technology (Lee and See, 2004; Cofta, 2009; Eiband et al., 2019; Glikson and Woolley, 2020; Meske and Bunde, 2020). Implementing XAI methods within HCI design for systems that include AI as decision support will help users become more aware of a system’s behavior and support a richer collaboration between humans and AI (Gilpin et al., 2018). Similarly, XAI research is also critical in domains such as transportation, finance, security, legal, and medicine, where AI decisions can potentially impact human lives.

Improving congruence between algorithmic decisions and human perceptions concerning those decisions is a serious challenge within advanced and critical machine learning applications (Doshi-Velez and Kim, 2017; Rudin, 2019). However, the complexity, recursivity, and high degree of nonlinearity of current machine learning systems make it arduous to dissect the decision process and, thus, provide straightforward and understandable explanations for a human to process. To tackle this challenge, current approaches in XAI create post-hoc algorithmic and mathematical methods to help explain initially opaque models. Moreover, researchers in the field have recently begun to investigate how to visually represent the AI decision process to produce intelligible explanations for humans (Sundararajan et al., 2019), highlighting the importance of visualization during the interpretation process.

According to a recent call for research (Abdul et al., 2018), a growing societal need is driving the rise in research interest investigating XAI. Furthermore, this call states that there exists a perception of bias in AI decision-making systems that affects both users of those systems and those for whom decisions are being made more generally. To address this need for research, we present a study investigating the effects of design choices of an AI model’s explanation visualization (EV) on the level of confidence between a human and an AI system. As a basis for the study presented here, we formulated the following research questions: “To what extent will the EV of an AI system’s decision affect a user’s confidence?”. And furthermore, to highlight if aspects of information presentation further affect user confidence, “To what extent does presentation order and visualization technique promote or degrade user confidence in the AI system and its decisions?”.

We present two studies designed to address these questions using Cognitive Fit (CF) as a theoretical basis and utilizing several EV methods of AI decisions output to manipulate morphological clarity (MC) and two presentation-order methods to investigate adjacency. The first study was administered online and assessed each EV’s impact while participants completed a simple decision-making task using an AI system’s outputs. The second study was performed using the same stimulus material and methodology in a laboratory. However, in this study, we recorded measures of pupillometry to infer cognitive load. Both studies investigate how user confidence in future system predictions is affected by each type of EV.

## Previous Work

### Trust and Confidence in Artificial Intelligence

It has been proposed that control or perception of control moderates confidence, whereby increasing confidence acts cumulatively to build trust in alliances and partnerships (Das and Teng, 1998). Trust in a specific technology, such as an AI system, affects the value-added proposition of using the technology after its adoption (Mcknight et al., 2011). Moreover, a user who has high confidence in a technologies capabilities and thus places trust in a specific technology is more likely to explore and use its features (Mcknight et al., 2011). Therefore, it has become essential to consider the trust relationship between user and technology when developing and implementing an AI system in order to accelerate its acceptance within the workplace. This relationship has a few proven antecedents, such as navigational structure and visual appeal, ease of use, and the national culture of the user (Vance et al., 2008). Furthermore, trust in an AI system is also dependent on more specific factors such as privacy, security, reliability, stated and perceived accuracy, and transparency (Cofta, 2009; Fairclough et al., 2015; Yin et al., 2019; Glikson and Woolley, 2020).

As described in Lee and See (2004), trust within a human-AI partnership is the attitude that an agent, such as an AI system, “will help achieve an individual’s goals in a situation characterized by uncertainty and vulnerability.” In this form of partnership, insufficient trust placed in a system may result in distrust and disuse of the system, whereas too much trust may result in over-reliance in the system, whereby the AI takes away from human agency and the ability to make decisions. Depending on the scale, importance, and impact of the tasks and decisions taken by the AI system, misuses or disuses of the system by its users can lead to safety and profitability problems (Parasuraman and Riley, 1997; Lee and See, 2004). While trust mediates how much reliance humans are willing to place on AI systems, trust also mediates how much humans rely on each other (Lankton et al., 2015). However, humans lose confidence in AI systems more rapidly than other humans, even if both parties make the same mistake (Dietvorst et al., 2015). Thus, it can be stated that trust in AI systems tends to decrease rapidly as a function of the number of errors the system makes over the duration of interaction and that the restoration of trust in AI systems or tools requires an undetermined but more significant amount of time (Glikson and Woolley, 2020; Nourani et al., 2020). Moreover, studies have shown that humans display greater trust toward other human agents than AI agents, even though both perform the same tasks and make identical mistakes (Jakesch et al., 2019; Glikson and Woolley, 2020).

However, according to (DeCamp and Tilburt, 2019), it is erroneous to speak of a user-AI trust relationship. Referring to trust in AI as a concrete psycho-affective relationship would imply that the system belongs to the same category of human agents that can be trusted, such as a physician or member of law enforcement. Currently, for an AI system, the thoughts, motives, and actions, which comprise the human psycho-affective framework involved in developing and allocating trust, go beyond its technical and mechanical capacities. Furthermore, moving forward, the development of AI will have a significant moral impact in the long term as the performance of AI improves, given that human capacities, in terms of technical accuracy, speed, and judgment, may well prove inferior to that of AI systems, resulting in a blanket of distrust regardless of evidence proving their accuracy and worth.

Moreover, some researchers suggest a game-theoretic approach where it is only possible to talk about trust when the trustor is in a situation of vulnerability and uncertainty with the trustee and when the consequences of a betrayal of trust are more significant than the benefits sought (Luhmann, 2001; Vance et al., 2008). In the context of the current research study, it would therefore be more appropriate to speak of developing confidence rather than trust in AI systems. Confidence in AI can be defined as a measure of risk as to how sure users are that they received the correct suggestions by the AI system and if they consider the system to be reliable, i.e., the system consistently operates properly, functional, i.e., the system does what it is supposed to do, and helpful, i.e., the system provides adequate help for the users (Lankton et al., 2015; Wanner et al., 2021).

### Cognitive Fit Theory

Cognitive Fit theory (Vessey, 1991) offers a means to understand how design choices of AI decision-making visualizations affect human cognitive performance and potentially toward building confidence in the system. This theory proposes that congruence between the task and the structure of the representation of a problem in an individual’s mental model results in reduced cognitive effort and superior performance. The complexity and usefulness of this mental model are dependent on the user’s working memory capacity (Vessey and Galletta, 1991; Goodhue and Thompson, 1995; Adipat et al., 2011). Therefore, additional cognitive effort is required when there is a lack of fit between the task at hand and the information format used to complete the task (Vessey, 1991). The individual must mentally transform that information into a format that enables them to accomplish the task, resulting in reduced performance (Adipat et al., 2011). This relationship between CF and cognitive effort was demonstrated using neuroscientific methods (Nuamah et al., 2020).

Cognitive Fit theory was first proposed to assess the effect of numerical data presented in a tabular versus function format paired with a symbolic and spatial task on cognitive performance. This theory has been applied in several studies over the years, within other research domains, and using multiple other information presentation formats (Adipat et al., 2011; van der Land et al., 2013; Chen, 2017; Gillespie et al., 2018). CF theory can also be used to support the theoretical basis of this study, where numerical data are translated into visualizations using colors and shapes for human usage. In this respect, the visualization of the AI decision process can potentially help provide a stronger CF between the task and the information required to complete that task in human-AI collaboration contexts, where understanding the AI decision process is of importance.

The result of designing tasks using this process is greater efficiency and effectiveness, manifested as lower cognitive load, increased accuracy, and speed in problem-solving. Therefore, applying this process to the presentation and visualization of AI decision reasoning may significantly impact the level of confidence engendered between a human and an AI system. Moreover, a visualization method with a stronger CF would help users perceive the reasoning behind the AI system’s prediction, consequently helping to avoid over or lack of confidence resulting in a misuse or disuse of the AI system (Lee and See, 2004). Furthermore, results from a recent study using the same stimuli and AI algorithm (Hudon et al., 2021) reported a negative correlation between cognitive load and the level of confidence in the AI system resulting from the use of an EV. These results further support the assertion that improving the CF between the task and the EV type may reduce cognitive load and positively influence a user’s confidence in the system.

### Progress in Explainable Artificial Intelligence

Recent advances in XAI have made it possible to produce explainable models and learning methods, especially in image recognition systems that have developed multiple techniques (Barredo Arrieta et al., 2020). Techniques such as Grad-CAM (Selvaraju et al., 2020) and integrated gradient (Sundararajan et al., 2017) aim to show relationships between inputs and outputs, focusing on processing information in the models. Studies have investigated the potential effect of interpretability and explanations on humans. Moreover, it has been shown that transparency in a model helped users simulate the model’s prediction (Poursabzi-Sangdeh et al., 2018). Others indicated that visualizations have a considerable influence on the effectiveness of the explanations (Sundararajan et al., 2019), that they can help discriminate between classes more accurately, help reveal a model’s trustworthiness, and help identify biases in datasets or models (Gilpin et al., 2018; Selvaraju et al., 2020). However, it is unknown what impact these visualization techniques have on developing confidence between human and AI systems to our knowledge.

## Hypothesis Development and Research Model

**FIGURE 1:**
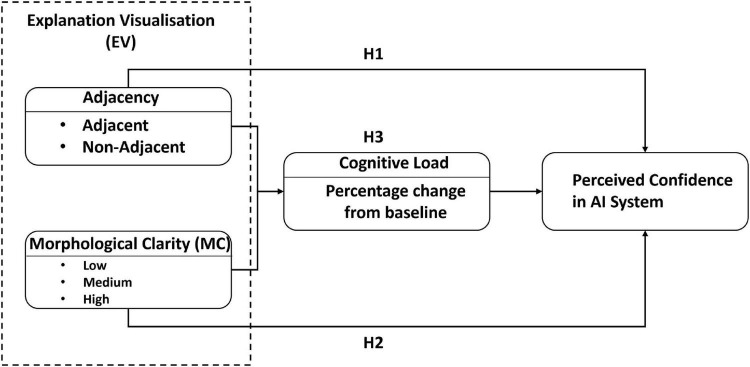
Research model.

The research model (Figure 1) posits that the design of an EV will affect a user’s confidence in the system. More precisely, we hypothesize that the effect of EV adjacency and MC, in addition to the interaction between both, will affect cognitive load influencing perceived confidence. The rationale behind these relationships is developed below.

Adjacency is a form of presentation order in which an adjacent EV is presented with its explanation data displayed directly upon the original image by coloring the areas in different colors to indicate their data values. In contrast, a non-adjacent EV is presented with the same explanation data but separated from the image (Dennis and Carte, 1998). However, non-adjacent EV requires greater cognitive effort to process since there is a loss in correspondence between the explanation and the visualization (Sundararajan et al., 2019). For spatial tasks, similar to the one used in the study presented here, (Dennis and Carte, 1998) showed that adjacent representations led to faster and more accurate decisions. They state that adjacent visualizations should help decision-makers associate areas of a visualization image with its data, simplifying the complex relationship between them, leading to faster, more accurate decisions. We posit that this adjacency relationship will hold for newer spatial tasks in which the visualization and data presented represent decisions already made by an AI and where the user of the AI is asked if he has confidence in future AI classification abilities.

Furthermore, we theorize that MC, which represents the degree to which a visualization displays clear delimited features by adjusting the appearance or removing specific data (e.g., noise) to help make the delimitation clearer for the user (Sundararajan et al., 2019), will also affect user confidence in the AI system. This study uses three levels of MC (i.e., low, medium, and high). First, low MC EVs are faithful to the model’s behavior as they allow the user to precisely identify at a pixel level what areas of the image are relevant. However, this precision makes the EV more cluttered, preventing the user from having a clear overarching view of the image. Alternatively, High MC EVs are more faithful to the MC definition by having clear form features while reducing the noise due to excess information. High MC EV provides greater clarity for the user and tends to be easier to comprehend by reducing his cognitive load but at the cost of faithfully depicting the model’s behavior. On the other hand, low MC EV might cause humans to misuse or ignore the explanation altogether by giving them too much information to process (Sundararajan et al., 2019; Müller et al., 2021). This concept of information overload (i.e., too much available information or too much high-quality information) has been shown to decrease the user’s decision effectiveness and can even make the user less confident about their decision (Keller and Staelin, 1987; Müller et al., 2021).

As discussed previously, CF theory (Vessey, 1991) proposes that the level of congruence between task and information presentation mediates task performance. Such that, while solving a problem, an individual creates a mental representation of the problem based on the information presented. This mental representation’s complexity and usefulness are dependent on the user’s working memory capacity (Vessey and Galletta, 1991; Goodhue and Thompson, 1995; Adipat et al., 2011). Thus, additional cognitive effort is required in the presence of incongruence between task and the format of the information presented to help complete the task (Nuamah et al., 2020), requiring the individual to mentally transform that information into a format suitable for accomplishing the task, resulting in reduced performance (Adipat et al., 2011).

We hypothesize that specific EVs will result in a better CF, reducing cognitive load, defined as the demand imposed by a task on the user’s working memory (Wickens, 2008). Therefore, a task requiring significant mental resources is more likely to prompt more user errors than a task requiring less cognitive resources, resulting in less perceived effort and greater CF (Palinko et al., 2010b; Nuamah et al., 2020).

Thus, we seek to determine whether *Adjacent EVs will result in higher confidence in the AI system* (H1) or if *High* MC *EVs will result in higher confidence in the AI system* (H2) or whether *the combination of adjacent and high* MC *EVs mediated by cognitive load will result in higher confidence in the AI system* (H3).

## Materials and Methods

### Study One

#### Experimental Design

We designed an online experiment to test our hypotheses. Specifically, we used a 2 × 3 within-subject factorial design to examine the effects of adjacency and MC of AI EV on confidence between the user and the AI system. The first factor considers the adjacency of the representation with two levels: EV with (adjacent) or without (non-adjacent) image background, the second factor considers the MC of the EV with three levels: low-cloud of points (CP), medium-heatmap (HM), and high-outline (ON). We considered CP EV to be low MC since it faithfully depicts the model’s attributions by highlighting the image’s pixels that positively impact the model’s classification. However, CP also displays much information that can be useless to the user. We considered HM EV to be a low MC since it is less precise than CP, showing only the model’s focal point on the stimuli image. HM also does not delimit its shapes enough to be considered a High MC visualization. Finally, ON EV draws only the most essential zones of the image used in the classification, therefore providing a high MC, but at the cost of pixel-level precision. See Table 1 for EV examples and the Generation of Explanations section for detailed information about these factors’ implementation.

**TABLE 1:** Overall inter-rater agreement for each group (G#) at each round (R#).

		Kappa		Raw agreement		Fleiss’s K		Krippendorff’s alpha		*N*	
		R1	R2	R1	R2	R1	R2	R1	R2	R1	R2
G1	J1 – J2	0.829	0.969	75.40%	78.80%	0.816	0.854	0.81	0.855	*N* = 65	*N* = 66
	J1 – J3	0.752	0.798								
	J2 – J3	0.858	0.798								
G2	J1 – J2	0.938	0.85	84.80%	82.10%	0.896	0.853	0.896	0.855	*N* = 66	*N* = 67
	J1 – J3	0.907	0.848								
	J2 – J3	0.845	0.863								
G3	J1 – J2	0.845	0.907	72.70%	89.40%	0.803	0.927	0.804	0.927	*N* = 66	*N* = 66
	J1 – J3	0.752	0.891								
	J2 – J3	0.814	0.984								
Average		0.838	**0.879**	77.63%	**83.43%**	0.838	**0.878**	0.837	**0.879**		

*Values in bold represent the final average across rater groups.*

#### Participants

Amazon Mechanical Turk (MTurk) was used to recruit participants. Based on (Jia et al., 2017) recommendations the participant pool was screened for North American residency to prevent linguistic difficulty. In the study, we restricted ourselves to local explanations of the model as they are deemed to be accessible for novices. This selection criterion avoids confounding factors due to participants’ potential machine learning expertise. A total of 350 North American participants took part in the survey, of which 206 (Avg. age = 37.87 ± 10.51, 123 Male) provided usable data. Sixty-one participants considered themselves AI or Data experts based on criteria posited by Mohseni et al. (2018). To improve data quality, data were removed for participants who failed any of the two attention checks, did the survey multiple times, or did not correctly submit their survey. The attention checks verified participant attention to the stimuli by displaying black squares instead of the images. Our institution’s ethics committee approved the study, and each participant provided informed consent and was compensated $CAD 20 upon completion.

#### Experimental Procedure

**FIGURE 2:**
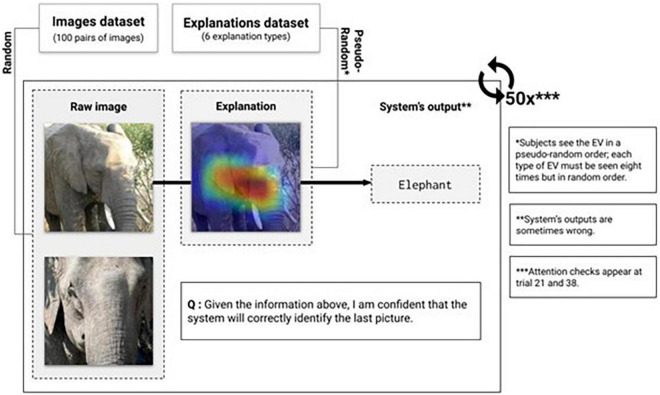
Task’s design. Images selected from the ImageNet dataset (Deng et al., 2009).

Once recruited, participants were directed to an online survey on Qualtrics. Participants were provided instructions and an example of a trial task upon beginning the study. For each trial, several elements were presented on screen (Figure 2): (1) The image analyzed by the system, (2) the system’s EV, (3) the system’s output, and (4) a related but unclassified image. In this case, the system’s output is the classification of the image predicted by the system; classifications were presented irrespective of correctness. Classification mistakes were evenly distributed among all EV types. For our 206 participants, we found on average per EV type 350 classification mistakes (STD = 10.61) compared to 1,259 correct classification (STD = 14,15). Participants were then asked to rate their agreement with the following statement using a 7-point Likert scale ranging from “Strongly disagree” to “Strongly agree”: “Given the information above, I am confident that the system will correctly identify the next picture.” The decision-making task consisted of 50 trials per participant, with two trials consisting of attention checks.

Stimulus material was presented randomly, with one label associated with a pair of similar images. In this context, similar images belong to the same class, therefore having the same label (e.g., two images of a car). A participant could not see the same pair of images more than one time. EV types were also presented in a random order for each task trial. The combination label-explanation type was also randomized amongst participants to avoid confounding effects due to the image label, object, or prediction. Therefore, a participant could see the image of an elephant paired with an adjacent-medium MC EV, and another could see the same image paired with a non-adjacent-low MC EV. After 50 trials, all six different EV types were seen and judged eight times by the participant. After completing the main task, participants were asked to answer demographic questions regarding their age, gender, and education level.

### Study Two

#### Experimental Design

For the second study, we designed a 2 × 3 within-subject factorial design to investigate the effects of adjacency and MC of AI-EVs on the user’s cognitive load and perceived confidence. The first factor considers the representation’s adjacency with two levels: EV with (adjacent) or without (non-adjacent) image background. The second factor considers the MC of the EV with three levels: low-CP, medium-heatmap (HM), and high-outline (ON). CP faithfully depicts the model’s attributions by highlighting pixels of the image that positively impact the model’s classification but also displays a high amount of superfluous information. HM is less precise than CP in terms of classification feature granularity. However, it shows the stimuli image’s prime focus used to make a classification but does not have precisely delimited features. The ON visualization draws only the most essential zones of the image used in the classification but at the cost of pixel-level precision. CP and ON-EVs were both implemented using the Integrated Gradients method (Sundararajan et al., 2017, 2019), and the HM-EV using the Grad-CAM class activation function (Selvaraju et al., 2020).

#### Experimental Procedure

**FIGURE 3:**
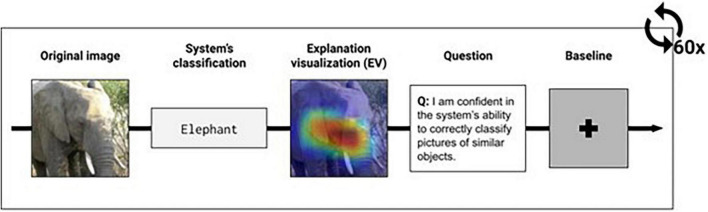
Task design. Images selected from the ImageNet dataset.

Nineteen participants (24.9 ± 8.3 years old, 10 Male) took part in the study, all signed consent and were compensated $CAD 30 upon completion. The study was approved by the ethics committee of our institution, the task consisted of a spatial task repeated over 60 randomized trials. Each trial involves a series of elements displayed on screen (see Figure 3) in the following order: (1) original image (e.g., image of an elephant), (2) classification of the image given by the AI system^1^ (e.g., “Elephant”), (3) the AI EV (e.g., an overlay IA explanation onto the original image), and (4) a perceived confidence question (e.g., confidence in the system).

Participants were asked to rate their agreement with the following statement using a 7-point Likert scale ranging from “Strongly disagree” to “Strongly agree” and “I am confident in the system’s ability to classify pictures of similar objects correctly.” A baseline image was finally shown at the end of each trial for 1 s. Each participant saw all six types of visualizations ten times. To measure pupil dilation, we used the Tobii ×60 eye tracker.

### Illuminance Testing

We measured lux using an Arduino light-dependent resistor (photoresistor) housed in a custom 3-D printed 1.5 cm depth × 5 cm area housing. Lux is a standardized unit of measurement of light intensity where 1 lux is equal to the illumination of a one-meter square surface that is 1 m away from a single candle. The experiment took place in a light-controlled room with an ambient lux value of lx (19.97). Luminance measurements for the display screen and per condition images were taken from a 70 cm distance and a height of approximately 1 m (representing a seated participant of average height). The default screen brightness at idle was lx (1.59), with the baseline fixation image lx (1.59). The average lux values per experimental condition were as follows: CP Adjacent lx (1.612), CP Non-adjacent lx (1.607), HM Adjacent lx (1.609), HM Non-adjacent lx (1.61), ON Adjacent lx (1.611), and ON Non-adjacent lx (1.67). We performed a 1-way ANOVA to determine if a significant difference existed between lux values that may affect pupillometry measures, no significant difference in luminance values between conditions was found *F*_Welch_ (5, 24.637) = 0.710, *p* = 0.621.

### Calculating Cognitive Load

Changes in Pupil dilation when a user faces a task requiring a high cognitive effort are referred to as the Task-evoked pupillary response (Beatty, 1982). We used pupil diameter to estimate the user’s cognitive effort required to process each EV. We computed the average percentage change from a baseline taken from a neutral image (PcB) for each participant and each EV in this analysis. We used the percentage change of pupil diameter rather than the raw pupil size variation due to inter-participant variance (Attard-Johnson et al., 2019).

### Selection of Stimuli

Subsets of stimulus images were selected from the ImageNet dataset (Deng et al., 2009), a publicly released image dataset with 1.2 million quality-controlled categorized images and associated human annotations. When building the stimulus image dataset, neutrality and unambiguity were used as the criterion. With these criteria in mind, straightforward categories of image and annotation pairs such as “Dog” instead of “Golden Retriever” or “Elephant” instead of “African elephant” were chosen to reduce confusion and present images as “platonic” classes. Image categories were chosen based on (Snodgrass and Vanderwart, 1980), who defined a standardized set of 260 illustrations of different concepts. These concepts were chosen based on three criteria: (1) They are unambiguously picturable, (2) they include exemplars from the widely used category norms of Battig and Montague (1969), and (3) which represent concepts at the basic level of categorization. As described by Battig and Montague (1969), unambiguity is the degree to which subjects will show consensus about the name to give the object. From these 260 concepts, 100 were selected to represent the categories of the images used within the stimulus dataset.

ImageNet organizes images according to the WordNet hierarchy (Miller, 1995), where each concept is described by one or multiple names called a “synonym set” or “synset.” The following criteria were established to select the stimulus images: (1) The image’s synset must contain the name of one of the 100 previously selected concepts, (2) No human subject is present in the image, and (3) the image must be neutral (no shocking or disturbing depiction). In total, 200 images were selected, giving two images per concept. Image ambiguity, familiarity, and complexity were measured and used as control variables in the final analysis.

### Validation of Stimuli

To ensure that the selected images are an unambiguous representation of the concepts outlined by Snodgrass and Vanderwart (1980), we used a panel of 18 judges to validate the images used for stimulus presentation. To ensure that three judges independently coded each stimulus image, the validation process was split into two rounds, and judges were split into six groups of three, thus there were nine judges in both rounds, and each group of three judges validated the entire stimulus corpus. As part of the evaluation process, each judge was asked to complete an online survey that displayed images to a screen and then write a label composed of one or two words to describe the image. Each group of three judges would assess between 65 and 68 images. This ensured that three judges provided labels for all images. Furthermore, judges were prompted to write a spontaneous description to minimize subjective bias. Following guidelines presented by Snodgrass and Vanderwart (1980), for each picture, the judges could also specify if: (1) They did not know the object (DNO), (2) if they knew the object but did not know its name (DKN), (3) and if they knew the name of the object, but it was momentarily irretrievable [tip of the tongue (TOT)].

In order to assess if a judges description (label) could be accepted or rejected, we specified the following criteria: (1) It is the same as the concept name (expected: bee, label: bee), (2) it is a synonym of the concept name (expected: aeroplane, label: plane), (3) it is a more precise term than the concept’s name (expected: bear, label: polar bear), and (4) it is listed in the nondominant list of name of the concept (Snodgrass and Vanderwart, 1980; expected: alligator, label: crocodile). An image was replaced if it matches one of these criteria: (1) At least two out of three judges put a rejected label or checked DKO, DKN, or TOT, or (2) at least two out of three judges use a nondominant name to describe the image.

The first round of stimuli verification was composed of three groups of three judges who produced an average Cohen’s Kappa of 0.838 (Table 1), which is considered a strong agreement rate (Viera and Garrett, 2005). However, eight images were replaced that did not meet the level of agreement criteria. We conducted a second round of stimuli validation to include the new images and nine new judges, which produced an average Cohen’s Kappa of 0.879 (Table 1). We did not replace any images after this second round of verification, given the high rate of inter-rater agreement.

### Choice of the Algorithm

To classify the stimulus images and act as the automated system in the study, we chose the Xception (Chollet, 2017) algorithm, which is packaged with pre-trained weights trained on the ImageNet dataset. Developed by a team at Google, Xception is a deep learning algorithm that relies heavily on prior effort done in the area of Convolutional Neural Networks. Xception has proven to be a very effective, compact (88 MB), and accurate (0.790) algorithm for computer vision problems (Chollet, 2017). We applied the trained Xception algorithm to the validated stimulus image. Output classifications, in the format of a sysnet-ID and label, were then compared to the “platonic” class of image. For example, if we are looking for the platonic class of “Cat” and the algorithm returned the synset label of “Cat” or “Tiger Cat,” we considered the classification acceptable as they both represent the class of a cat. After training, we obtained a reasonable classification rate of 0.76, close enough to the algorithm’s current maximum accuracy rate.

### Generation of Explanation Visualizations

In order to actualize the concepts of adjacency and MC during the experimental task, we utilized several visualization and presentation methods to provide the participant with an AI EV. The low and high MC visualizations were both implemented using the Integrated Gradients method (Sundararajan et al., 2017, 2019). These visualizations highlight in green the areas of the stimulus image that positively impact the model’s classification. The medium MC visualization was implemented using the Grad-CAM class activation function (Selvaraju et al., 2020). This method displays a heatmap that highlights activated regions important for the classification of the image.

**FIGURE 4:**
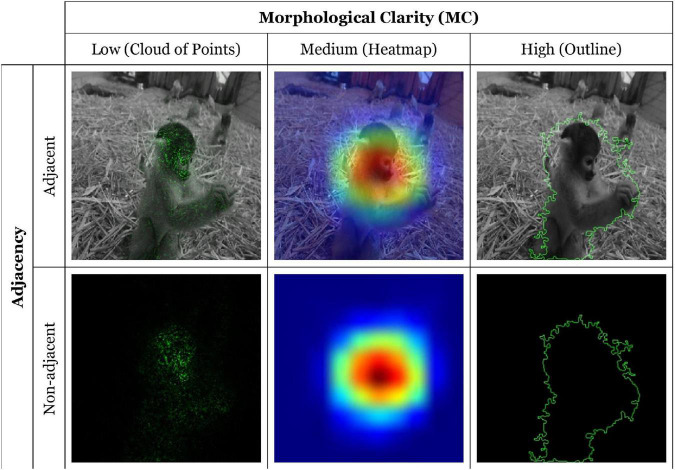
Example of each type of EV for the classification “Monkey”. Images selected from the ImageNet dataset (Deng et al., 2009).

Adjacency representations were implemented using the Grad-CAM class activation visualization overlaid upon the original image and the Integrated Gradients visualization overlaid upon a grayscale version of the original image, ensuring that the attribution colors did not blend with those of the image as recommended by Sundararajan et al. (2019). Non-adjacent representations were implemented by showing a black background under each visualization. In total, six combinations of EV per image were generated (Figure 4).

### Analysis

Using SAS 9.4, we performed a repeated-measures ANOVA (Bonferroni corrected) for the dependent variable Perceived Confidence (PConf) with both Adjacency, MC, and the interaction of those two as within-subject factors. PConf is a discrete variable taking values from 1 to 7, where 7 represents the highest value of confidence between the user and the system. Adjacency is a categorical binary variable (i.e., adjacent and non-adjacent), and MC is a categorical, ordinal variable (i.e., low, medium, and high). Since a classification’s validity can significantly impact a user’s perceived confidence in the system, we used the variable “Classification” as a covariate in the model. Classification is a Boolean variable indicating whether the model’s classification is correct.

In addition to the analysis performed in Hudon et al. (2021), for study two, we computed a within-subject, repeated measures mediation analysis using Monte Carlo methods (Yzerbyt et al., 2018), in *R* (JSmediation) to estimate the confidence interval of the indirect effect. Mediation analysis tests whether an IV affects a DV through a third variable, the mediator, in this case, cognitive load.

## Results

### Study One

The results show a statistically significant main effect of Adjacency [*F*(1, 205) = 246.96, *p* < 0.001], and MC [*F*(2, 410) = 5.69, *p* = 0.004]. Additionally, we observed a significant interaction between MC and Adjacency [*F*(2, 410) = 6.10, *p* = 0.003]. The covariate variable Classification also has a main effect on confidence [*F*(1, 205) = 768.13, *p* < 0.001].

*Post hoc* comparisons of simple effects (Table 2) reported that adjacent EV (*M* = 5.01, SD = 0.06) results in higher confidence than non-adjacent EV [*M* = 4.55, SD = 0.06; *t*(205) = 15.71, *p* < 0.001], providing support for H1. Concerning MC, *post hoc* comparisons showed that a low MC (Cloud of Points) EV (*M* = 4.85, SD = 0.06) resulted in higher confidence than both medium MC (Heatmap; *M* = 4.77, SD = 0.06; *t*(410) = 2.36, *p* = 0.019), and high MC (Outline; *M* = 4.73, SD = 0.06; *t*(410) = 3.27, *p* = 0.001), providing no support for H2, as the reported results highlight that the effect is the opposite of what we had hypothesized. The comparison between good classifications (*M* = 5.27, SD = 0.06) results in higher confidence than bad classification [*M* = 4.29, SD = 0.06; *t*(205) = 27.72, *p* < 0.001].

**TABLE 2:** *Post hoc* comparisons adjacency, MC, and classification on confidence.

Factor (1)	Factor (2)	Mean diff (1–2)	DF	*t*	*p*
Adjacent	Non-adjacent	0.46	205	15.71***	<0.001
Low MC (Cloud of points)	Medium MC (Heatmap)	0.08	410	2.36*	0.019
Low MC (Cloud of points))	High MC (Outline)	0.12	410	3.27**	0.001
Medium MC (Heatmap)	High MC (Outline)	0.03	410	0.91	0.363
Good classification	Bad classification	0.98	205	27.72***	<0.001

*p < 0.05, **p < 0.01, and ***p < 0.001.

**FIGURE 5:**
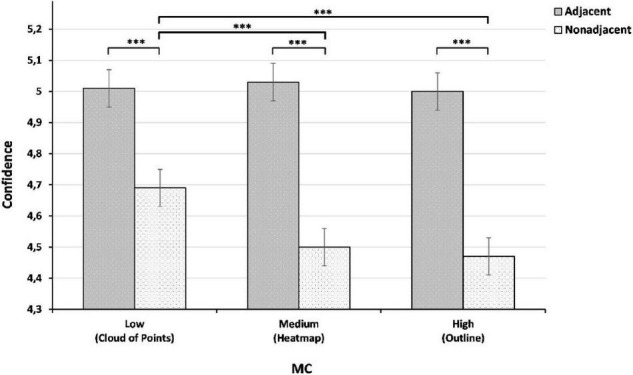
Comparison of adjacent and non-adjacent visualizations based on their MC from low to high. ***p < 0.001.

As shown in Table 3, adjacent-high MC EV did not significantly differ from the two other adjacent representations. Therefore, H3 is also not supported. Surprisingly, a non-adjacent-low MC EV (*M* = -0.25, SD = 0.09) resulted in a significantly greater positive impact on confidence than both non-adjacent-medium MC EV, and non-adjacent-high MC EV. Figure 5 illustrates that all three adjacent EV types have a significantly greater positive impact on confidence than their non-adjacent equivalent. It also shows that low MC EV has a significantly greater positive impact on trust than medium and high MC EV.

**TABLE 3:** *Post hoc* comparisons of the interaction between adjacency and MC on confidence.

Adjacency	MC level (1)	MC level (2)	Mean diff (1–2)	DF	*t*	*p*
Adjacent	Low (Cloud of points)	Medium (Heatmap)	–0.024	410	–0.48	0.632
	Low (Cloud of points)	High (Outline)	0.009	410	0.18	0.857
	Medium (Heatmap)	High (Outline)	0.033	410	0.66	0.511
Non-adjacent	Low (Cloud of points)	Medium (Heatmap)	0.191	410	3.82***	<0.001
	Low (Cloud of points)	High (Outline)	0.223	410	4.44***	<0.001
	Medium (Heatmap)	High (Outline)	0.032	410	0.63	0.530

***p < 0.001.

### Study Two

**FIGURE 6:**
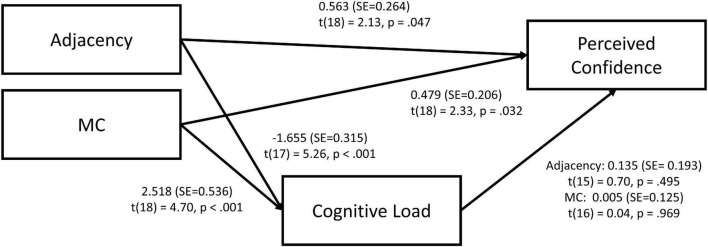
Mediation model for adjacency and MC on perceived confidence *via* cognitive load.

Preliminary results from the second study were published in (Hudon et al., 2021). These results reported a significant main effect of adjacency (*p* < 0.001) and MC (*p* < 0.001) and a significant interaction between both factors (*p* < 0.001) upon perceived confidence. However, in this preliminary analysis, any cognitive load mediation effect was never considered. Shown in Figure 6 are the results from a mediation analysis that follows the research model proposed above (Figure 1).

While the direct effect of adjacency on cognitive load was significant [*t*(17) = 5.26, *p* < 0.001], the indirect effect on perceived confidence [*t*(18) = 2.13, *p* = 0.47] *via* cognitive load is not, showing no indirect mediating effect of cognitive load on perceived confidence. Congruent with the above results, the Monte Carlo (5000 bootstrap samples) confidence interval for the indirect effect for adjacency reported no indirect effect upon perceived confidence and cognitive load (CI95% [–0.885; 0.408]).

Similarly, for MC, while the effect of MC on perceived confidence [*t*(18) = 2.33, *p* < 0.032] and cognitive load [*t*(18) = 4.17, *p* < 0.001] is significant, the effect of cognitive load on perceived confidence is not [*t*(16) = 0.04, *p* = 0.969], showing a lack of indirect effect. Monte Carlo confidence interval for MC reported no indirect effect upon perceived confidence and cognitive load (CI95% [–0.615; 0.655]).

**FIGURE 7:**
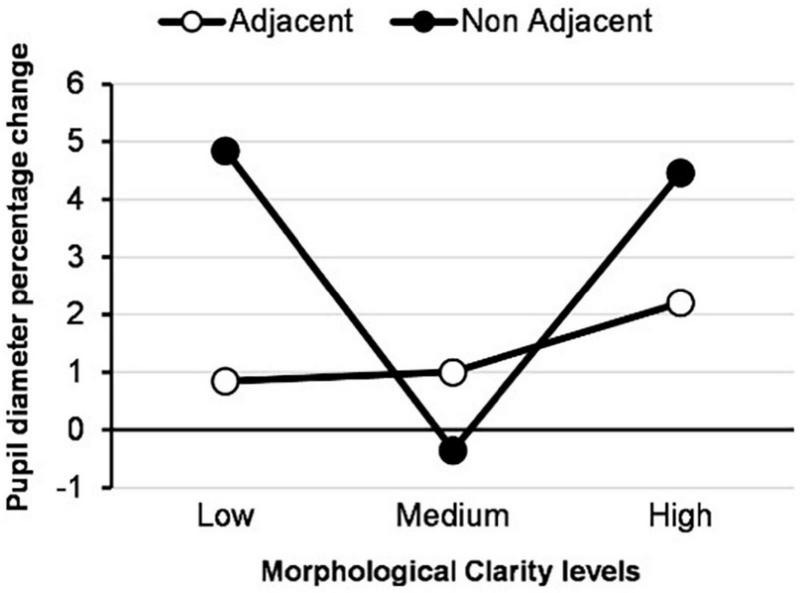
Percentage change of pupil diameter by each type of EV.

**FIGURE 8:**
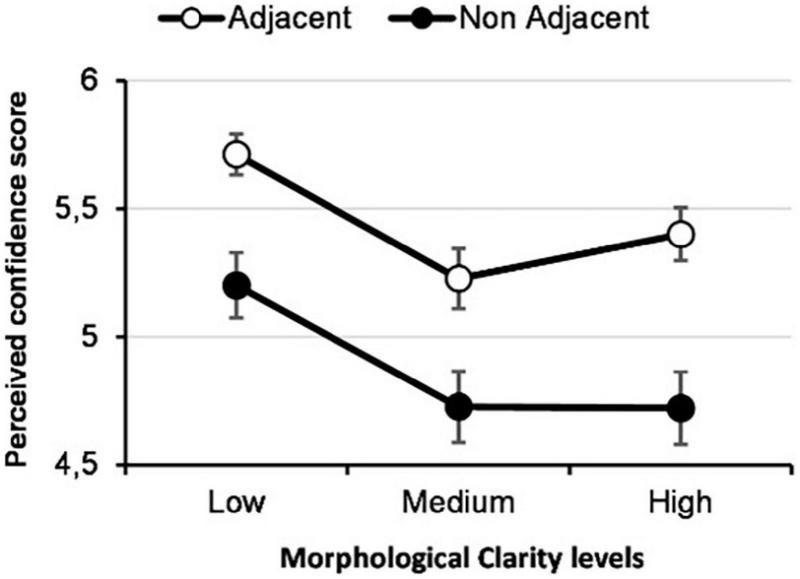
Perceived confidence for each type of EV.

These results indicate that the pathway to confidence *via* cognitive load as a mediator (H3) is not supported. However, the effects of both adjacency and MC have a very strong influence on the level of perceived confidence; and furthermore, each presentation method strongly influences cognitive load (see Figures 7, 8).

## Discussion

Overall, the results from these studies indicate a clear difference when comparing EV types, such that adjacency strongly influences perceived user confidence. Looking at this significant difference in more detail, we found that adjacent EVs had a more significant positive effect on user confidence than non-adjacent EVs, confirming our hypothesis. However, contrary to our initial postulation in which we surmised that a high MC would provide more than sufficient information and accuracy of representation to influence user confidence positively, low MC EVs resulted in the highest confidence between all three levels of MC. Furthermore, looking at the results from the interaction of both adjacency and MC, the findings indicate no clear method of information presentation, in that all three adjacent EVs have an approximately equivalent impact on user confidence. Moreover, the results indicate that in situations of non-adjacency, there is a significant difference between low and medium MC in addition to low and high MC, where the latter resulted in lower confidence in the system. Results from study 2 confirm these results. Finally, and perhaps counterintuitively, cognitive load decreases as MC increases with adjacent visualizations, with low MC showing the lowest cognitive load while this representation shows the highest density in terms of information and noise.

### The Impact of Adjacency on User Confidence

We first hypothesized that adjacent visualizations would have a greater significant impact on user confidence than non-adjacent EVs, as adjacency is posited to provide a superior CF with the task at hand in terms of providing visual scan patterns for pertinent information, potentially leading to higher confidence through a reduction in epistemic uncertainty. The results indicate, at least in part, that our hypothesis holds true and that adjacency does indeed impact user confidence significantly. This effect can be explained by the lower cognitive effort required to identify the correspondence between the highlighted areas of the explanation and the original image. Users were able to promptly identify which areas of the image influenced the AI system in its decision, helping them compare AI classification reasoning with their own. Indeed, (Dennis and Carte, 1998) posited that adjacent visualizations led to more accurate decision-making in less time when the user is faced with a spatial task, resulting in a better CF. However, non-adjacent EVs have the advantage of aiding the user in inspecting the details of the explanation without the distraction of the underlying image or reviewing the original image to verify what the attributions highlight in order to form a fresh opinion (Sundararajan et al., 2019). Moreover, non-adjacent EVs ask the user to continuously gaze back and forth between the original image and the EV. Thus, in terms of CF, we suggest that users need to mentally associate the original image zones with the AI decision visualization, creating a more complex mental map requiring more cognitive resources.

### The Conditional Effect of Morphological Clarity on User Confidence

We further hypothesized that high MC visualizations would have a more significant positive effect on user confidence. This visualization technique focuses and highlights important regions of the classified images, reducing the signal-to-noise ratio related to the EV representing the network’s results, thus diminishing the information load required. However, our results surprisingly indicate the opposite to be true, showing in this case that low MC EV had a more significant positive effect on user’s confidence than high and medium MC EVs. An alternate interpretation for this relationship could be that low MC EV’s include more information, even if that additional information would ordinarily be considered noise. Furthermore, this supplementary information may implicitly increase the perception of transparency in the model and positively affect user confidence. This interpretation aligns with (Brunk et al., 2019), who showed that the interpretability of black-box algorithms is perceived to be more transparent and trustworthy if additional information is present.

However, the adjacency comparisons (Figure 5) indicate no significant difference between levels of MC when explanation data are presented adjacently. As can be seen in the table, in our case, the main significant variance between levels of MC is between non-adjacent low MC with high and medium MC EVs. These results imply that low MC EV has a greater positive effect on confidence but only in situations of non-adjacency. With these conditions in mind, and in light of our interpretations, the results strongly suggest that users require more information about the explanation when adjacency requirements are not met. We interpret this difference between non-adjacent MC levels to be a perceptual and processing disassociation between EV and the subject matter of the input image. Moreover, while participants could make a certain sense of the target objects form in the case of the non-adjacent-low MC EV, the non-adjacent-medium and high MC EVs were too abstract to identify clear forms and associate them with the input image. As shown in Figure 4, the non-adjacent-low MC EV allows the shape of the monkey’s head to be distinguished, contrary to the two other non-adjacent EVs. Therefore, we cannot conclude overall that low MC EV has a greater effect on user confidence.

Overall, the results appear to indicate that in a task context requiring no precise knowledge (i.e., identifying a simple object in an image), confidence in an AI system can be improved with explanations, providing there is adjacency between the EV and the original image and furthermore, that the EV highlights areas of the original image that correspond to the user’s perceptual understanding of the task, regardless of the EV’s level of precision. In contrast, when users with greater knowledge in a particular subject are tasked with identifying specific patterns requiring precision (e.g., detecting diabetic retinopathy), they are much more critical about the type and characteristics of the EV used (Sundararajan et al., 2019). However, further research is required to investigate the relationship between the type of task and the explanations provided to complete that task in more detail.

### The Mediating Effect of Cognitive Load on User Confidence

Our previous analysis (Hudon et al., 2021) indicated that adjacent EVs resulted in lower cognitive load. We posited that this implied that the cognitive effort required to process and understand the AI’s EV through adjacent presentation is significantly lower than non-adjacent EV, given a reduced need to mentally associate the EV with the original image as the association is already made implicit within the EV itself. This effect was hypothesized by Dennis and Carte (1998), who stated that combining adjacent presentation order with a spatial task may lead to faster and more accurate decision making, resulting in a better CF. Furthermore, we reported low cognitive load for non-adjacent-medium MC EV. We posited that because this type of EV is very abstract, it potentially makes it difficult to process the original image’s target object and allows for a snap judgment and lower cognitive workload through a disengagement effect.

However, as the current analysis indicates, it is primarily adjacency and MC that act as mediators toward increased user confidence in the AI decision visualizations and not the level of cognitive load directly. Overall, in terms of CF, the combination of low MC and non-adjacency results in the highest perceived confidence for non-adjacent visualizations even under conditions of high cognitive load. We previously proposed (Hudon et al., 2021) that the high density of information presented in this EV potentially helped users identify the target object forms by reducing epistemic uncertainty. In that, the extraneous but useful information allowed the user to swap from cognitive processing to perceptual processing to understand the model’s behavior. Ongoing research in human factors is investigating the concept of epistemic uncertainty and how it affects man-machine teaming (Tomsett et al., 2020) in complex systems.

The results from the mediation analysis further indicate that the pathway to increased confidence *via* cognitive load as a mediator (H3) is not supported in our case. However, the effects of both adjacency and MC appear to have a strong direct influence on the level of perceived confidence, and that each presentation method significantly influences cognitive load. This result opens new questions for research concerning the mechanisms of action and the relationship between MC, adjacency, and perceived confidence in the results of AI decision making, which may impact how these systems are created and utilized by end-users.

### Decision-Making Dynamics: Situation Awareness, Ecological Interface Design, and Fast and Slow Thinking

Speaking in terms of decision-making dynamics, another complementary perspective from which to view these results may be through the combination of the model situation awareness (SA) proposed by Endsley (1995) ecological interface design (Bennett and Flach, 2019) and “fast and slow thinking” (Kahneman, 2011). When combined, these three approaches provide a dynamic model that blends design, perception and mechanisms that may explain the effects of MC and adjacency with regard to AI decision visualizations upon the user.

Situation awareness was defined by Endsley (1995; ref pp 1) as “the perception of the elements in the environment within a volume of time and space, the comprehension of their meaning, and the projection of their status in the near future,” the model thus consists of three tiers: perception, comprehension, and projection, where each tier within the model details the human factors associated with a dynamic system’s current and future state of information. The overarching goal of ecological interface design is to transform the behaviors required for workers to do their jobs: from activities requiring the worker to draw upon limited-capacity cognitive resources to activities that allow powerful perception and action capabilities to be leveraged. A critical process in achieving this goal is to understand the abstract, complex, semantic structure of a work domain and then design graphical displays that make it both concrete (i.e., easy to see) and meaningful (i.e., easy to interpret) to the worker.

Kahneman (2011) proposed that the human decision-making process is split into two parts a “fast” system and a “slow” system or system one and system two. System one includes two variants of intuitive thought, the expert and the heuristic, and operates at relatively fast speeds and automatically with providing no sense of voluntary control drawing upon the automatic neural systems involved with perception and memory. This system is the primary reactive system that consequently biases decisions *ex ante* based on a coherent interpretation of reality given current knowns and unknowns. System two by contrast, operates at a slower rate and at the subconscious level to allocate attentional resources to effortful mental activities and the conscious level to engage reasoning and volition such as when performing calculations or consciously attending to a problem. In terms of overall functionality and versatility, system two has the broader range in terms of decision making, however, it is system one that is dominant in the majority of situations as it is tied to many autonomic sensory and cognitive functions.

Thus, using this perspective to interpret the results reported here, the sense of SA creates task specific expectations within the user as it builds during task completion, these expectations are influenced by the elements of the interface design, in the current case the various visualizations of AI decisions. The developing sense of SA and the interactions with the interface dynamically cohere toward a more complete sense of SA related to the task, mediated by the adjacency and MC of information presentation within the interface. Therefore, the effects of MC and adjacency speak to methods of creating ecologically valid interfaces that positively affect SA. These further engage the decision-making process at various levels where the combination of both fast and slow processes mediated by adjacency and MC aggregate toward an increase in trust and confidence, i.e. if a task is repetitive requiring a quick “instinctual” decision, design interfaces with adjacency as a focus which engenders confidence and trust in the system until an error occurs. If, however, a more considered decision is required, such as a diagnosis from medical imagery for example, then design with non-adjacency, low MC and moderate cognitive load as a focus, to engage more rational processes through evidence gathering and consonance with expected outputs (building toward good SA) which engenders confidence building through perceived collaborative effort where errors are shared between system and user.

### Contribution to Theory and Implications for Practice

The theory of CF has been used in previous research as a framework to explain the impact of different information presentation methods paired with various tasks on decision-making performance. Studies from various fields have provided validation of this theory, such as computer system development (Shaft and Vessey, 2006), geographic information systems (Dennis and Carte, 1998), and e-commerce (Brunelle, 2009; Chen, 2017). Our study incorporates CF theory into XAI by assessing which combination of presentation method and type of EV provided a better “fit” and positively influenced confidence using a spatial task. The results reported in this study provide evidence showing that adjacent EVs facilitate the CF of a problem and its representation for the user, in that they help users effectively utilize their working memory while performing the task by presenting the information in a more “understandable” and structured format than non-adjacent EVs.

Studies have shown that visualized explanations of a system or model positively impact confidence (Eiband et al., 2019; Meske and Bunde, 2020) and that the presence of transparent design further increases this impact (Kizilcec, 2016; Weitz et al., 2019). We add to this design template for confidence by showing that the choice of EV type in terms of its adjacency and MC may also modulate the impact on user confidence. Furthermore, designs that impede the level of CF between interpretability and the task, such as in the case of non-adjacent EV, may have a null or even negative impact on confidence compared to cases where there is no explanation transparency. Implying that not all explanations of AI decision processes, be they visual or not, positively impact user trust in AI decision systems. Therefore, when designing AI decision tasks and interpretability interfaces, the aim should be to achieve a high level of congruence between the problem-solving task and the problem representation to align with the user’s mental representation and knowledge of the task.

## Limitations and Future Work

The current study did not consider the cognitive distance or the degree of similarity between two images representing the same label. Consequently, some pairs of images may have appeared similar (e.g., two images of an elephant’s face) and others rather different (e.g., dogs of different breeds). Users presented with an AI system’s classification could potentially be more confident that the system will correctly classify a similar image than if they are presented with a dissimilar image. This difference in similarity between pairs of images could result in a bias in the results. Therefore, future work should control the degree of similarity between pairs of images. We see two opportunities to address this challenge. Firstly, one should adopt an algorithmic perspective and compute the similarity between pictures and secondly, one could task a new panel of judges to assess the perceived visual distance between images.

In this study, we only performed an analysis on the effect of Adjacency and MC on the user’s confidence without including the sample’s demographic data in the model. These data could have a moderating effect on the user-AI confidence (e.g., the confidence of an AI expert might be less influenced by the different EV representation than an AI novice). Moreover, we did not consider the potential impact on the confidence of the individual’s propensity to trust AI. Some people are more likely to trust technology than others, and it would have been interesting to see the extent of this effect on confidence.

Finally, we did not control for algorithm type and design in this study. Further work should concentrate on replicating our results for different algorithms to examine if our observations generalize. It has been demonstrated that even if the explanation algorithm is model-agnostic, intrinsic differences in the image recognition system design can lead to differences in predictive features (Ribeiro et al., 2016). Thus, future research could investigate the impact of design choices in black-box models on the interpretability explanation and its impact on the human-algorithm interaction.

While most of the work on interpretability focuses on algorithmic approaches to create interpretable models, this study shows that human-centered EVs of such algorithms can provide an avenue for new research, where explanations are designed to fit the users’ capabilities and the context of use. Future studies could investigate the impact of the highlighted areas of an image to determine if the highlighted areas that show a similar thinking process to humans have a greater impact on confidence than others.

Furthermore, explainability as currently recognized by the explainable AI community, highlights technically relevant parts of machine representations and machine models, i.e., those elements that have contributed to model accuracy (as both negative and positive attribution in the current study). However, this form of explainability does not make reference to a human-readable model which is potentially a limitation of the current approach. As a consequence, researchers introduced the term “causability,” defined as cause identifiability, as an extension to “cause suitability” as a potential measurable model for human interpretability (Holzinger et al., 2020). Utilizing this model as a means through which to measure explainability would allow the measurement of how an explanation of a statement reaches a specified level of causal understanding for a user, in which the effectiveness, efficiency and satisfaction of an explanation can be determined for a given context of use. As previously discussed and further highlighted by researchers in the field (Holzinger and Müller, 2021), this will require new human-AI interfaces in the future that enable contextual understanding and also allow domain experts to ask questions and counterfactuals, i.e., “what if” questions. Investigating this new model and form of measurement is something to be considered moving forward toward explainable AI.

Concerns related to the lack of confidence between humans and AI are not only relevant to the field of HCI. They have far-reaching societal implications, as AI systems take critical decisions that can impact human lives. To continue developing better collaboration between human and AI agents, there is a need to focus on developing interpretable, transparent models that present information in a way that is compatible with human cognitive processes (European Commission, 2021). In this regard, research in the field could be expanded to include the concurrent use of neurophysiological assessment methods such as eye-fixation related potential (Palinko et al., 2010a). Studying the active brain while testing new interpretation methods would add an extra dimension that allows the testing of CF against the cognitive load of adjacent and non-adjacent low, medium, and high MC visualizations of new interpretation methods.

## Conclusion

This study investigated the relationship between various types of EV of an AI system’s output and user confidence in the system’s decisions. The results show that visualization and information presentation design choices have the potential to positively impact a user’s confidence in the AI system. By drawing upon CF theory (Vessey, 1991), we show that in this case, adjacency and the level of MC of the visualization have a conditional relationship upon user confidence. An EV overlaid upon the original image, no matter the level of precision of the visualization, results in a better fit between the decision task and the explanation resulting in higher confidence. On the other hand, users showed a preference for visualizations providing precision in situations of non-adjacency of the EV.

Furthermore, the relationships between various types of EVs used to explain an AI system’s output and users’ cognitive load cannot be explained simply as a function of cognitive load, while visualization presentation methods do contribute to both greater and lesser cognitive load, cognitive load in and of itself does not mediate the effect on a user’s confidence in system decision output. The results indicate that design choices related to EVs can positively impact a user’s confidence in AI systems by reducing epistemic uncertainty. Overall, our results strongly suggest that the careful consideration and application of CF theory, adjacency methods and EVs containing low MC to AI interface and task design may help improve the confidence relationship between a user and an AI decision support system. Most of the advances on interpretability and machine learning are still model-centric; this research proposes a human-centered approach to evaluate interpretability design choices.

## Data Availability Statement

The raw data supporting the conclusions of this article will be made available by the authors, without undue reservation.

## Ethics Statement

The studies involving human participants were reviewed and approved by HEC Montreal Research Ethics Board (REB). The patients/participants provided their written informed consent to participate in this study.

## Author Contributions

AK: ideation, coordination, design and development, analysis, interpretation, and writing. TD: ideation, design and development, implementation, architecture, and analysis. AH: experimental design and procedures and analysis. P-ML: supervision, insight, and editing ideation. SS: ideation, insight, and supervision. All authors contributed to the article and approved the submitted version.

## Conflict of Interest

The authors declare that the research was conducted in the absence of any commercial or financial relationships that could be construed as a potential conflict of interest.

## Publisher’s Note

All claims expressed in this article are solely those of the authors and do not necessarily represent those of their affiliated organizations, or those of the publisher, the editors and the reviewers. Any product that may be evaluated in this article, or claim that may be made by its manufacturer, is not guaranteed or endorsed by the publisher.

## Footnotes

1 The Xception (extreme inception; Chollet, 2017) algorithm which comes with pre-trained weights on the ImageNet dataset was used to classify the images.
